# Secondary metabolic profiles and anticancer actions from fruit extracts of immature pomegranates

**DOI:** 10.1371/journal.pone.0255831

**Published:** 2021-08-10

**Authors:** Venera Russo, Alberto Continella, Carmelo Drago, Alessandra Gentile, Stefano La Malfa, Claudia Giovanna Leotta, Luana Pulvirenti, Giuseppe Ruberto, Giovanni Mario Pitari, Laura Siracusa

**Affiliations:** 1 Vera Salus Ricerca S.r.l., Siracusa, Italy; 2 Dipartimento Agricoltura Alimentazione ed Ambiente (Di3A), Università di Catania, Catania, Italy; 3 Istituto di Chimica Biomolecolare del CNR (ICB-CNR), Catania, Italy; Institute for Biological Research, University of Belgrade, SERBIA

## Abstract

Immature fruits from *Punica granatum* L. thinning are a neglected side product of pomegranate production with cumbersome disposal costs for farmers. To explore value potential of immature fruits from pomegranate ‘Wonderful’ cultivars, the compositional landscapes and antitumorigenic activities of pomegranate extracts from two different stages of maturation were assessed. Cancer cell proliferation and cytotoxicity was quantified in human lung H1299 and colon HCT116 adenocarcinomas by crystal violet staining, MTS assay and caspase-3 activity. High performance liquid chromatography—diode array detector (HPLC/DAD) and high performance liquid chromatography—electrospray ionization—mass spectrometry (HPLC/ESI-MS) analyses indicate that immature fruits are rich sources of gallotannins and ellagitannins, with the highest amounts contained in immature fruit peels. Biological investigations reveal a robust anticancer activity by those immature *P*. *granatum* fruit extracts, which reflected induction of tumor cytotoxicity and cell death mechanisms. Together, present observations suggest *P*. *granatum* byproducts from the thinning process may provide unexplored values for virtuous circular economy.

## Introduction

Pomegranate (*Punica granatum* L.) is one of the most popular and studied fruit of recent decades. A great interest for this natural matrix resides in the peculiar biological properties of both its juice [1] and side products, mainly peels [2–4]. Peel, seeds and mesocarps obtained during pomegranate juice production have been extensively investigated [5, 6]. However, other pomegranate byproducts such as those originating from the fruit thinning process have received scarce attention. In this context, pomegranate extracts from ripe fruits exhibit promising antitumor activities in different cancer types [7–9], particularly against lung and colorectal adenocarcinoms [10, 11], the top two cancer killers worldwide [12]. Anticancer effects by pomegranates have been ascribed to various metabolic constituents, including polyphenols [13] and polysaccharides [14, 15]. Biological mechanisms underlying tumor inhibition are also diverse, spanning from suppression of cancer growth, angiogenesis and metastasis to induction of cell cycle arrest and apoptosis [16]. Whether similar considerations may be extended to immature pomegranate fruits remains unclear.

Previous observations from nine different *P*. *granatum* cultivars suggest byproducts from fruit thinning, the removal of flowers/clusters of flowers or individual fruitlets after pomegranate fruit set, could represent rich sources of bioactive compounds [17]. In pomegranate cultivation, the period of full bloom lasts about 1 month and fruit set occurs in 2 to 4 distinct periods, with high quality fruits obtained in the early bloom [18]. When excessive fruit set occurs, fruit thinning is recognized to improve fruit size and quality, and to promote bloom return in coming years [19, 20]. However, the removal and disposal of immature fruits during the thinning process often require deployment of costly waste management procedures, representing undesirable economic burdens for local producers. Therefore, valorization of byproducts from the pomegranate thinning process through novel, virtuous paradigms of circular economy is warranted.

**Fig 1:**
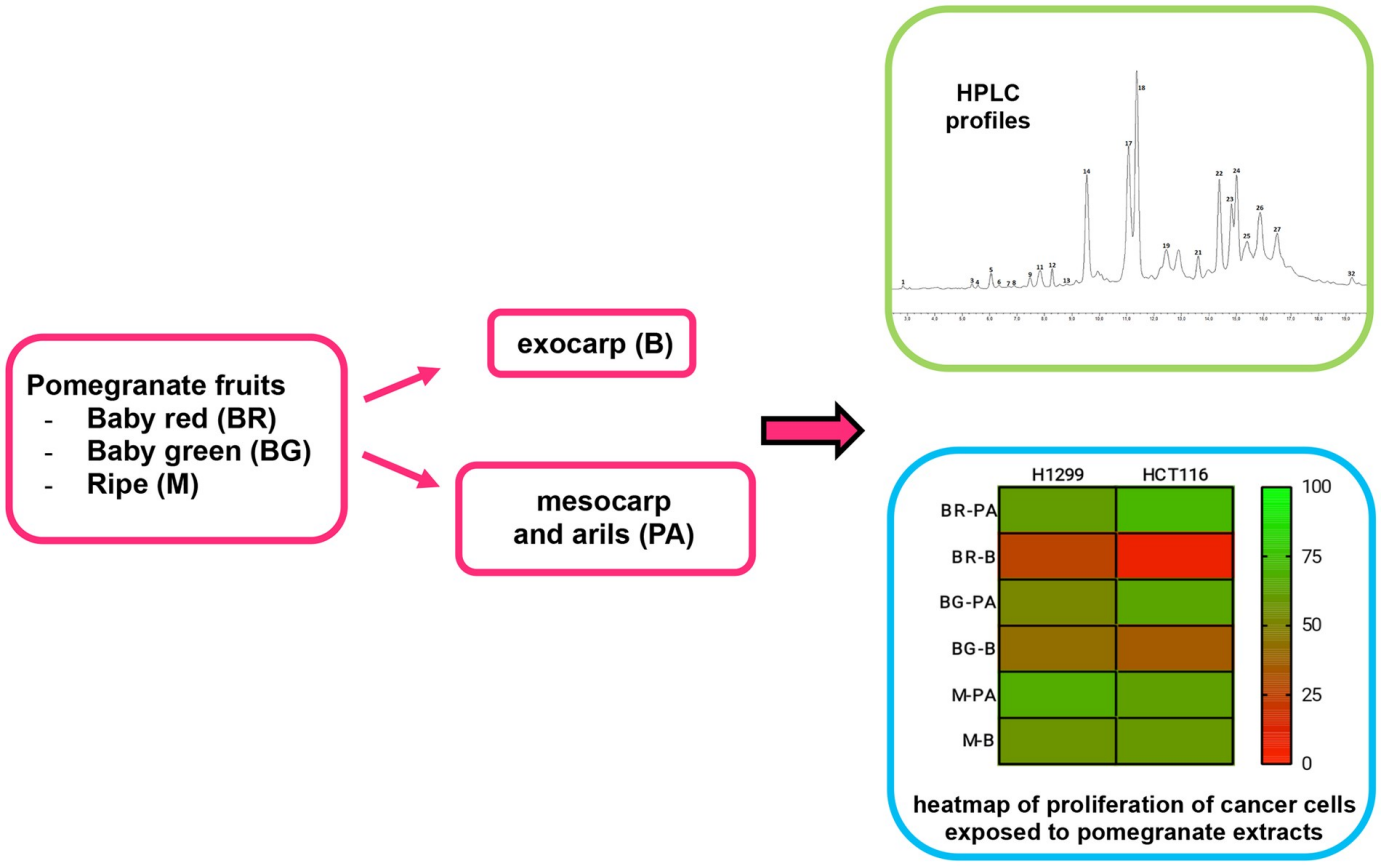
Workflow scheme of the studies performed.

Here, detailed compositional profiles of immature ‘baby’ fruits from ‘Wonderful’ cultivars, obtained from thinning procedures at two different stages of maturation, are reported for the first time. Immature fruits were separated into mesocarp/arils and peels to characterize metabolic composition and bioactive properties. Ripe pomegranate extracts were employed as reference controls. The anticancer potential of these matrices in the context of human lung and colon tumors was investigated with two distinct immortalized cancer cell lines. The experimental workflow of these studies is summarized in Fig 1.

## Materials and methods

### General

All chemicals and solvents were of analytical grade and used without further purification. Pure reference standards gallic acid, ellagic acid and punicalagin were purchased from Sigma Aldrich (Milan, Italy). HPLC grade water and acetonitrile were from VWR (Milan, Italy). Human lung adenocarcinoma H1299 and human colorectal carcinoma HCT116 cells were obtained from the American Type Culture Collection (ATCC; Manassas, VA, USA). Cell line authentications were provided by the vendor. Dulbecco’s modified Eagle’s medium (DMEM), RPMI 1640 medium, fetal bovine serum (FBS) and 0.25% trypsin solution were from Euroclone S.p.A. (Pero, Milan, Italy). Cells were maintained at 37°C and 5% CO_2_ in media supplemented with 10% FBS, 100 units/mL penicillin and 100 μg/mL streptomycin (Invitrogen, Carlsbad, California, USA). Cells were used between passages 5–15.

### Plant materials

Fruits from 7 years old trees of a ‘Wonderful’ pomegranate orchard in Marsala (Trapani, Italy; 37°52’12”N, 12°29’36”E) were a gift from a local consortium of pomegranate producers. Trees were planted at 3.5 × 6 m spacing and cultivated accordingly to standard practices for commercial use. At around 5 and 9 weeks (or 35 and 60 days, respectively) after fruit bloom onset, fruit thinning was performed on 10 uniform, randomly selected trees (5 trees at each time) by manually removing axillary fruits on clusters. Immature fruits from earlier thinning were labelled ‘baby red’, while those obtained at the latest time ‘baby green’. Mature fruits were also collected at the time of harvest (in October 2018) from uniformly selected trees and made available for subsequent investigations. All fruits collected were immediately stored (at 4±1°C) until further processing.

### Extraction of pomegranate matrices and sample preparation

**Fig 2:**
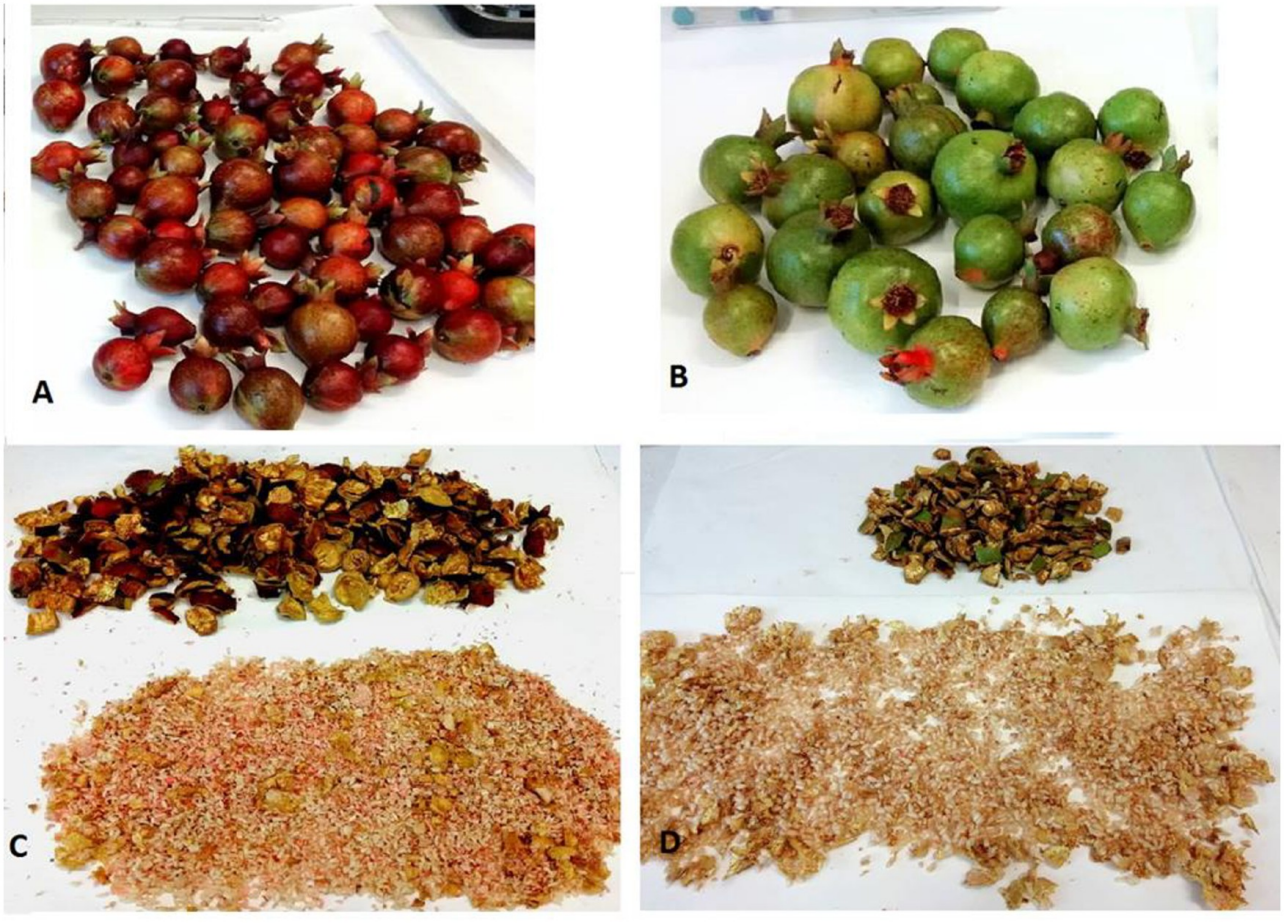
Immature pomegranate ‘baby red’ (A, C) and ‘baby green’ (B, D) as whole fruits and divided into peels and mesocarp/arils ready for extractions.

Immature (baby red, BR; baby green, BG) and ripe (M) pomegranate fruits were accurately washed and dried with sorbent paper. After manually separation of leathery peel (exocarp, B) from fleshy mesocarp and arils (PA; Fig 2), 100–200 g each of these fruit matrices were suspended in 400–500 mL of a weakly acidic hydro-alcoholic solution (MeOH: H_2_O:HCO_2_H, 80:19:1 v/v) and minced *in situ* with an electric hand blender. The resulting heterogeneous suspensions were left stirring overnight at room temperature (RT) on magnetic plates in the dark, and then filtered under vacuum on *buchner* funnel equipped with moistened standard laboratory filter paper to recover the coloured mother solutions. Samples (1–2 mL) from those solutions were opportunely diluted to obtain concentrations of 5–12 mg fresh material/mL that were immediately analyzed by HPLC. The remaining amounts of the solutions were lyophilized (Lyoquest-85, Telstar Italy, Legnano, Milan, Italy) and stored in a dark and dry place until use. With this procedure, the following freeze-dried extracts were obtained:

$$\begin{array}{cccc} \text{fruit}\;\text{matrix} & \text{Code} & \begin{array}{c} \text{weight}\;\text{after}\;\text{freeze-dying} \end{array} & \begin{array}{c} \text{yietd}\;\text{from}\;\text{fresh}\;\text{fruits} \end{array}\\ \text{baby}\;\text{red}\;\text{pomegranate}\;\text{mesocarp}\;\text{and}\;\text{arils} & \text{BR-PA} & 4.86\;\text{g} & 6.1\%\\ \text{baby}\;\text{red}\;\text{pomegranate}\;\text{peels} & \text{BR-B} & 10.31\;\text{g} & 12.8\%\\ \text{baby}\;\text{green}\;\text{pomegranate}\;\text{mesocarp}\;\text{and}\;\text{arils} & \text{BG-PA} & 4.67\;\text{g} & 5.8\%\\ \text{baby}\;\text{green}\;\text{pomegranate}\;\text{peels} & \text{BG-B} & 10.10\;\text{g} & 12.6\%\\ \text{mature}\;\text{pomegranate}\;\text{mesocarp}\;\text{and}\;\text{arils} & \text{M-PA} & 25.00\;\text{g} & 4.6\%\\ \text{mature}\;\text{pomegranate}\;\text{peels} & \text{M-B} & 23.00\;\text{g} & 23.0\% \end{array}$$

### HPLC/DAD and HPLC/ESI-MS analyses

Chromatographic analyses were carried out on an Ultimate3000 UHPLC focused instrument equipped with a binary high pressure pump, a Photodiode Array detector, a Thermostatted Column Compartment and an Automated Sample Injector (Thermo Fisher Scientific, Inc., Milan, Italy). Collected data were processed with a Chromeleon Chromatography Information Management System (v. 6.80). Analytical runs were performed using a reverse-phase column (Gemini C18, 250 x 4.6 mm, 5 μm particle size; Phenomenex Italia s.r.l., Bologna, Italy) equipped with a guard column (Gemini C18 4 x 3.0 mm, 5 μm particle size; Phenomenex Italia s.r.l., Bologna, Italy). Pomegranate metabolites were eluted using the following gradient of B (2.5% formic acid in acetonitrile) in A (2.5% formic acid in water): 0 min: 0% B; 25 min: 35% B; 30 min: 0% B. The solvent flow rate selected was 1 mL/min, the temperature 25°C and the injector volume 10 μL. Quantification of gallic acid and its derivatives (including HHDP derivatives, pedunculagins and granatins) was performed at 280 nm employing gallic acid (r_2_ = 0.9999) as the reference control. In contrast, quantification of ellagitannins and ellagic acid (and its derivatives) was performed at 360 nm using punicalagin (r_2_ = 0.9998) or ellagic acid (r_2_ = 0.9997) as reference controls, respectively. In order to unambiguously identify the chromatographic signals and to confirm peak assignments, HPLC/ESI-MS analyses were also performed. ESI mass spectra were acquired using the same equipments and methodology previously described [21]. All analyses were carried out in triplicate.

### Cell viability

Cancer cells were seeded (H1299, 1x10^3^; HCT116, 8x10^2^) in 96-well plates and grown for 72 h at their respective optimum culture condition. Then, cells were treated with 100 μg/mL of M-PA, M-B, BG-PA, BG-B, BR-PA and BR-B for additional 48 h. Control cells received an equal volume of vehicle (DMSO). At the end of incubations, cells were fixed (in 4% paraformaldehyde) and stained with crystal violet (1%, aqueous solution). For quantification, after crystal violet extraction with 10% acetic acid (at RT for 10 min) the absorbance (at 590 nm) was measured with a spectrophotometer (Synergy HT, BioTek).

### Cytotoxicity

Cytotoxicity was quantified with the CellTiter 96^®^ AQueous One Solution kit (Promega Corp., Madison, WI, USA), assessing cell metabolic fitness through MTS [3-(4,5-dimethylthiazol-2-yl)-5-(3-carboxymethoxyphenyl)-2-(4-sulfophenyl)-2H-tetrazolium, inner salt]. Briefly, cells (5x10^3^) were seeded in 96-well plates and left adhering for 24 h (at 37°C). Then, cells were treated for additional 24 h (in serum-free media) with 100 μg/mL of the indicated pomegranate extracts or the vehicle control (DMSO). At the end of treatments, 20 μL/well of the CellTiter 96^®^ AQueous One Solution Reagent was added, cultures incubated for 1 h (at 37°C) and the absorbance (at 492 nm) was measured with a spectrophotometer (Synergy HT, BioTek).

### Cell death

Cell death via apoptosis was assessed by quantifying caspase-3 activity with the Ac-DEVD-AMC fluorogenic substrate (Cayman Chemical, Ann Arbor, MI, USA). After seeding (H1299, 1x10^4^; HCT116, 5x10^3^) in 6-cm dishes and culturing for 24 h, BG-B and BR-B treatments (100 μg/ml, each) were started in serum-free media and continued for additional 24 h. Then, cells were lysed and cytosolic aliquots (20 μg proteins) incubated for 1 h with Ac-DEVD-AMC (20 μM) in the caspase-3 assay buffer. Upon substrate cleavage by the caspase-3 activity present in cell fractions, resulting amounts of free fluorescent AMC were quantified (absorbance at 440–460 nm) with a fluorescent plate reader (Synergy HT, BioTek). Data were normalized to protein contents and expressed as fold increase of caspase-3 activity relative to the vehicle control.

### Statistical analysis

Results are shown as mean ± SEM of either three independent experiments performed in quadruplicate (for cell viability and cytotoxicity assays) or six independent experiments performed in single replicate (for apoptosis assays). Multiple comparisons were conducted by One-way ANOVA with Dunnett’s post hoc test. P values were considered significant at α≤0.05. All statistical analyses were done with GraphPad Prism 8.0 (GraphPad Software, Inc., San Diego, CA).

## Results and discussion

### Secondary metabolic profiles of different parts of pomegranate immature and ripe fruits

**Fig 3:**
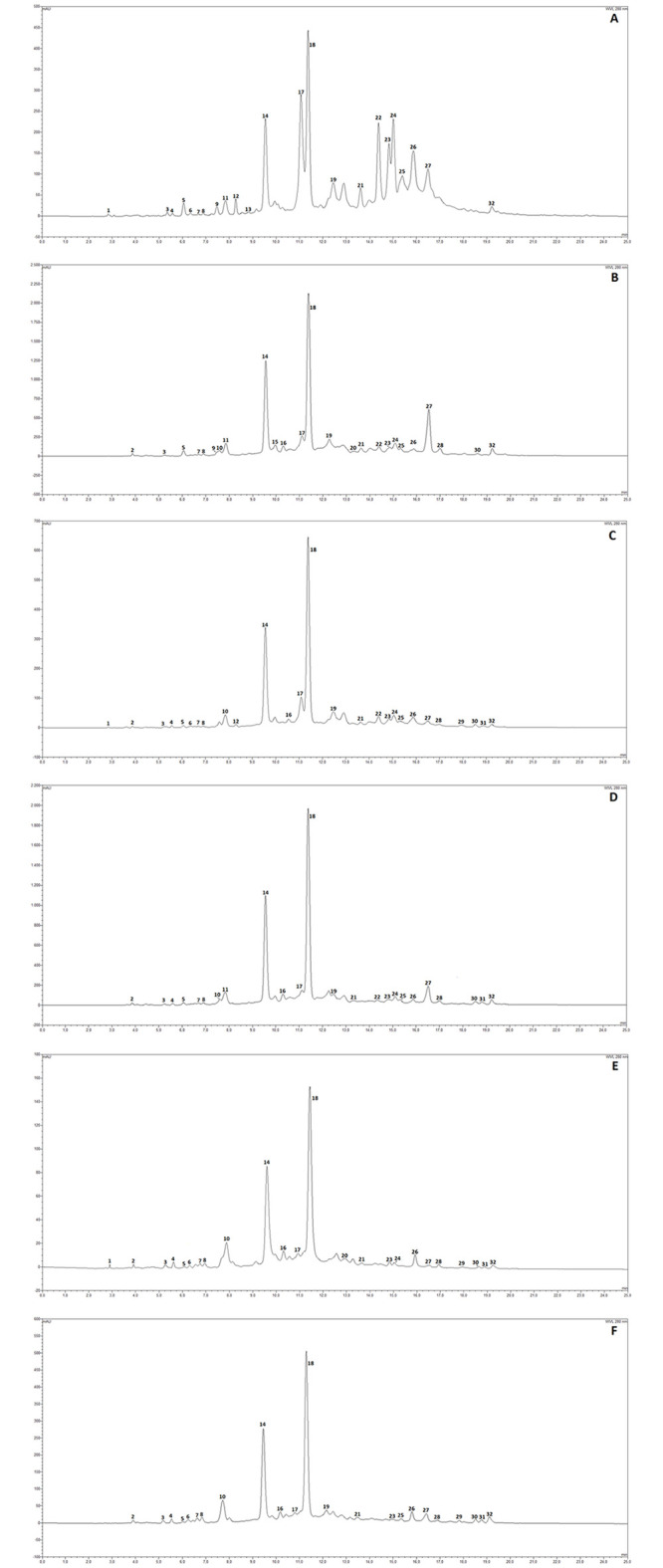
HPLC-DAD chromatograms of pomegranate matrices (at 280 nm). A) Extract from ‘baby red’ immature pomegranate mesocarp and arils (BR-PA). B) Extract from ‘baby red’ immature pomegranate peels (BR-B). C) Extract from ‘baby green’ immature pomegranate mesocarp and arils (BG-PA). D) Extract from ‘baby green’ immature pomegranate peels (BG-B). E) Extract from ripe pomegranate mesocarp and arils (M-PA). F) Extract from ripe pomegranate peels (M-B). See Fig 2 for matrix identification, Table 1 for numbering and Material and Methods for details.

The secondary metabolic profile and content of peel, mesocarp and arils from immature ‘baby’ pomegranate fruits at two different maturation stages were studied employing a series of HPLC/DAD and HPLC/ESI-MS analyses. The corresponding DAD chromatograms (visualized at 280 nm) are shown in Fig 3. Chromatograms E (mesocarp and arils) and F (peels) correspond to extracts from ripe pomegranate fruits, here used as controls.

A total of 32 peaks were detected and identified on the basis of relative retention times, UV-Vis and mass spectral data. Injection with pure analytical standards and comparisons with literature data corroborated the assignments. Pomegranate fruit is characterized by the presence of a multitude of hydrolysable tannins, with a peculiar mixture of anomers punicalagin a and b [21]. Gallotannins and ellagitannins are the most represented subclass of polyphenols, followed by simple ellagic acid derivatives. Gallotannins, ellagitannins and generally hydrolysable tannins from pomegranate originate from a very limited number of simple building blocks, including gallic acid (which gives rise to both hexahydroxydiphenyl HHDP and ellagic acid) and glucose. The almost countless number of chemical structures derived from the combination of these molecules constitute the metabolic pool of pomegranate [3, 22, 23], and were confirmed in present studies (Table 1 and S1 Fig). The UV-Vis spectra of the identified peaks allowed to discriminate gallotannins (19 peaks) from ellagitannins (tannins containing an ellagic acid moiety; 7 peaks) and 6 simple ellagic acid derivatives (Table 1, peak list and diagnostics). All these subclasses share almost identical UV-Vis spectra, and only mass spectrometric analyses by TIC (total ion current chromatograms) and EIC (extracted ion chromatograms) permitted rational identifications (Table 1). Qualitatively, chromatograms (Fig 3) appeared very similar between different extracts, with punicalagin a and b (peaks 14 and 18) as the main compounds in all fruit matrices. Exceptions include chromatographic analyses of BR-PA, corresponding to mesocarp and arils from ‘baby red’ fruits (Figs 2 and 3A), where peak 17 (pedunculagin, a bis-HHDP glucose derivative; S1 Fig) is nearly as abundant as punicalagin a, and peaks 21–26 (punigluconin and pedunculagin isomers; Table 1) are bigger than those in other chromatograms. Similarly, for BR-B (peels from ‘baby red’ fruit; Fig 2) peak 27 is unusually abundant and dominates the second part of the chromatogram (at 12–25 min; Fig 3B). Peak 27 has been identified as granatin B, a gallotannin bearing a peculiar enantiomeric dehydrohexahydroxydiphenoyl [24, 25]. Peak corresponding to granatin B is relevant also in BG-B, the peels from the ‘baby green’ pomegranate fruit (Fig 3D).

**Table 1:** Peak list and diagnostics of selected metabolites from pomegranate matrices object of this study.

Peak no.	Rt, min^a^	compound tentative identification	UV-vis data, nm^b^	MW	ESI^-^ data, m/z^c^
1	2.8	lagerstannin C (galloyl-HHDP-glucose)	238, 260sh	650	649 (M-H)^-^, 301*
2	3.8	HHDP-hexoside	258, 280 sh	482	481 (M-H)^-^, 345*
3	5.2	galloyl hexoside	279.4	332	331 (M-H)^-^
4	5.5	galloyl-HHDP- hexoside	278	634	633 (M-H)^-^, 301*
5	6.0	gallic acid^d^	280	170	169 (M-H)^-^
6	6,3	punicalagin derivative	269, 370	1124	1123(M-H)^-^, 1101
7	6.6	punicalin (gallagyl-hexoside)	259, 380	782	781(M-H)^-^, 601*
8	6.9	punicalin isomer	259, 381	782	781(M-H)^-^
9	7.4	di(HHDP-galloylglucose)-pentoside	243.4, 268.2	1416	1415(M-H)^-^, 783*
10	7.7	punicalagin isomer	243.8, 370	1084	1083 (M-H)^-^
11	7.8	pedunculagin isomer 1	243.3	784	783(M-H)^-^, 601*
12	8.2	galloyl hexoside isomer	280	332	331, 169*
13	8.7	digalloyl-hexoside	240, 272	484	483(M-H)^-^, 271*
14	9.4	punicalagin a^d^	357.9, 378.2	1084	1083 (M-H)^-^, 601*
15	9.9	punicalagin isomer	257, 378	1084	1083 (M-H)^-^
16	10.5	pedunculagin III	243.7	934	933(M-H)^-^, 721*
17	10.9	pedunculagin isomer 2	244.6, 264.6	784	783(M-H)^-^, 301*
18	11.3	punicalagin b^d^	257.9, 379	1084	1083 (M-H)^-^*, 601
19	12.2	punigluconin isomer	244.9	802	801(M-H)^-^, 347*
20	12.9	ellagic acid deoxy-hexoside	254, 362	448	447(M-H)^-^, 301*
21	13.6	Punigluconin	272.8	802	801(M-H)^-^, 649*
22	14.3	pedunculagin isomer 3	270	784	783(M-H)^-^, 301*
23	14.8	pedunculagin II	269.7	786	785 (M-H)^-^, 633*
24	15.0	pedunculagin isomer 4	271.6	784	783(M-H)^-^, 765*
25	15.3	pedunculagin isomer 5	271	784	783(M-H)^-^, 765*
26	15.8	ellagic acid hexoside	252, 360	464	463 (M-H)^-^, 301*
27	16.4	granatin B	278	952	951(M-H)^-^, 933*
28	16.9	granatin B isomer	278	952	951(M-H)^-^
29	17.8	ellagic acid galloyl-hexoside	253, 360	616	615(M-H)^-^, 301*
30	18.6	ellagic acid pentoside	253, 358	434	433 (M-H)^-^*
31	18.8	ellagic acid galloyl-hexoside isomer	253, 360	616	615(M-H)^-^*
32	19.2	ellagic acid^d^	252, 365	302	301(M-H)^-^*

^*a*^ as mean of six matrices x three replicates = 18 analyses;

^*b*^ from HPLC;

^*c*^ base peaks marked with an asterisk;

^*d*^ co-injection with pure commercial standard (refer to Fig 2 and text for details).

### Secondary metabolic content of different parts of pomegranate immature and ripe fruits

**Fig 4:**
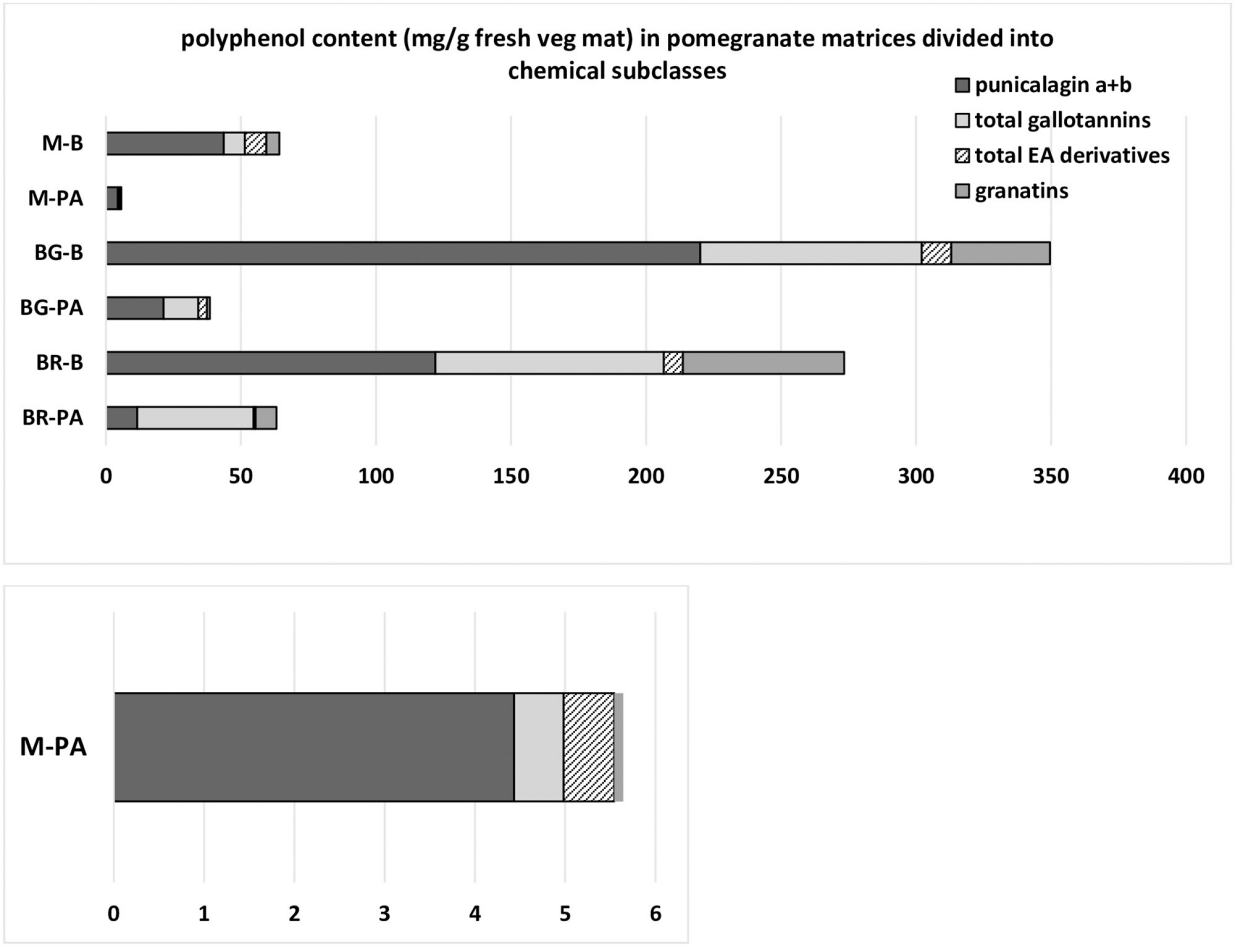
Polyphenol contents (mg/g of fresh vegetable material) of pomegranate matrices. Polyphenols were divided into chemical subclasses. BR-PA, BR-B, BG-PA, BG-B, M-PA and M-B correspond to extract labels as indicated in Fig 3. See also S1 Table for individual and collective quantitative data.

The selected metabolites identified in pomegranate products and byproducts were then divided into chemical subclasses and quantified as mg/g of fresh fruit materials (Fig 4 and S1 Table), to highlight the value potential of the pre-harvest waste products. BG-B (peels from ‘baby green’ immature pomegranates) resulted the richest extract in polyphenols, with nearly 353 mg polyphenols/g of fresh fruit material, followed by BR-B (peels from ‘baby red’ immature pomegranates) with 296 mg/g of fresh material. In both samples, the total amount of polyphenols accounted for 1/3 of the matrix fresh weight. The remaining extracts presented comparatively modest phenolic contents, with highest amounts in M-B (peels from mature pomegranates) containing 67.5 mg polyphenols/g of fresh material, and BR-PA (mesocarp and arils from ‘baby red’ immature pomegranates) with 63.2 mg/g of fresh material. It is noteworthy to note that the earlier removal of ‘baby red’ fruits, compared to that of ‘baby green’, has higher positive effects for fruit development, as it determines a greater resource availability and, consequently, increased fruit size [26, 27]. For all samples, the amount of polyphenols was higher in peels than in mesocarp and arils, as already reported [3]. The anomers punicalagin a and b were confirmed as the most abundant metabolites in all matrices except BR-PA, in which total gallotannin amounts were the highest (Fig 4 and S1 Table). Granatins (granatin B and its isomer, peaks 27 and 28, Fig 3) were confirmed to be particularly abundant only in BR-B (nearly 60 mg/g of fresh fruit material) and BG-B (36.5 mg/g of fresh material, S1 Table). As a general trend, a noticeable decrease in phenolic content was observed from immature fruits ‘baby red’ and ‘baby green’ to ripe pomegranates (S1 Table). This result is a very well documented phenomenon in pomegranate, which contributes to the astringency loss and sweet taste gaining of mature fruits [28, 29]. Interestingly, quantitative analyses (S1 Table) demonstrated an opposite trend from immature-to-mature pomegranate matrices in content amounts (%) of punicalagin a and b and total gallotannins over total phenolics (S2 Fig), as punicalagin anomers increased from BR (‘baby red’ fruit) to M (mature fruit) while total gallotannins decreased. These data corroborate the hypothesis of a different accumulation behavior during maturation for the distinct subclasses of pomegranate metabolites, a novel finding in the field.

### Anticancer effects of pomegranate immature and ripe fruit extracts

**Fig 5:**
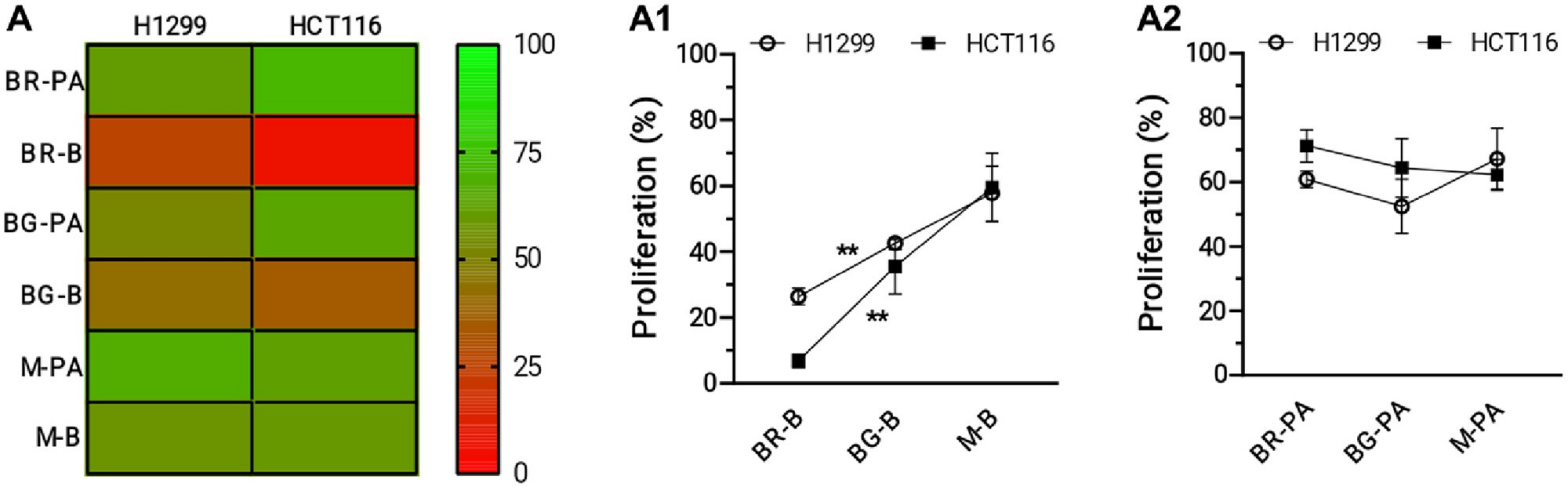
Anticancer effects of different extracts from pomegranate immature and ripe fruits. A) Heatmap of proliferation in H1299 and HCT116 cells exposed for 48 h to 100 μg/mL of the indicated pomegranate fruit extracts. Data (left) and color scale (right) reflects % of respective controls treated with the vehicle DMSO. A1) Trend analysis of anticancer effects by pomegranate peel extracts shown in A. **, p ≤ 0.01. A2) Trend analysis of anticancer effects by pomegranate mesocarp/arils extracts shown in A.

In order to define antitumor properties of immature pomegranate extracts in lung and colon cancers, the ability to inhibit cancer cell proliferation was investigated in human adenocarcinoma H1299 (lung) and HCT116 (colon) cells. Extracts (at 100 μg/mL, each) from different maturation stages significantly inhibited cancer cell proliferative kinetics compared to the vehicle controls (S3 Fig). However, antiproliferative activity was greatest in peels extracts from immature pomegranate fruits compared to all other parts (S3 Fig). In general, peel (B) extracts showed higher anticancer effects compared to respective mesocarp/aril (PA) counterparts (Fig 5A), probably reflecting differences in polyphenol contents between these two matrices. Notably, antiproliferative B-PA differences followed a ripening gradient (Fig 5A), wherein the greatest, significant difference was present in the baby red (BR) immature fruit and the smallest, not significant difference in the mature (M) fruit (Table 2).

**Table 2:** Statistical comparisons of antiproliferative activity of pomegranate immature and mature fruits treatments in cancer cells H1299 and HCT116^a^.

*Pomegranate extract*	H1299			HCT116		
	95.00% CI of diff	P Value	Significance	95.00% CI of diff	P Value	Significance
BR-B vs. BR-PA	14.53 to 54.44	0.002	**	44.51 to 84.42	<0.001	***
BR-B vs. BG-B	-36.17 to 3.745	0.11	ns	-48.69 to -8.772	0.007	**
BR-B vs. BG-PA	-46.07 to -6.154	0.01	*	-77.62 to -37.71	<0.001	***
BR-B vs. M-B	-51.34 to -11.43	0.003	**	-72.73 to -32.81	<0.001	***
BR-B vs. M-PA	-60.89 to -20.98	<0.001	***	-75.48 to -35.57	<0.001	***
BR-PA vs. BG-PA	-11.58 to 28.33	0.39	ns	-13.16 to 26.76	0.49	Ns
BG-B vs. BG-PA	-10.06 to 29.86	0.32	ns	8.983 to 48.90	0.006	**
BG-B vs. BR-PA	-1.680 to 38.23	0.07	ns	15.78 to 55.70	0.001	**
BG-B vs. M-B	-35.13 to 4.784	0.13	ns	-44.00 to -4.086	0.02	*
BG-B vs. M-PA	-44.68 to -4.769	0.02	*	-46.75 to -6.838	0.01	*
M-B vs. M-PA	-10.40 to 29.51	0.33	ns	-17.20 to 22.71	0.78	Ns
M-B vs. BR-PA	-16.85 to 23.06	0.75	ns	-8.260 to 31.65	0.24	Ns
M-B vs. BG-PA	-25.23 to 14.68	0.59	ns	-15.06 to 24.85	0.62	Ns
M-PA vs. BR-PA	-26.41 to 13.51	0.51	ns	-11.01 to 28.90	0.36	Ns
M-PA vs. BG-PA	-34.78 to 5.130	0.14	ns	-17.81 to 22.10	0.83	Ns

^*a*^ BR = “baby red” immature fruits; BG = “baby green” immature fruits; M = ripe pomegranate fruits; PA = mesocarp and arils; B = peels.

**, p ≤ 0.05;

**, p ≤ 0.01;

***, p ≤ 0.005.

**Fig 6:**
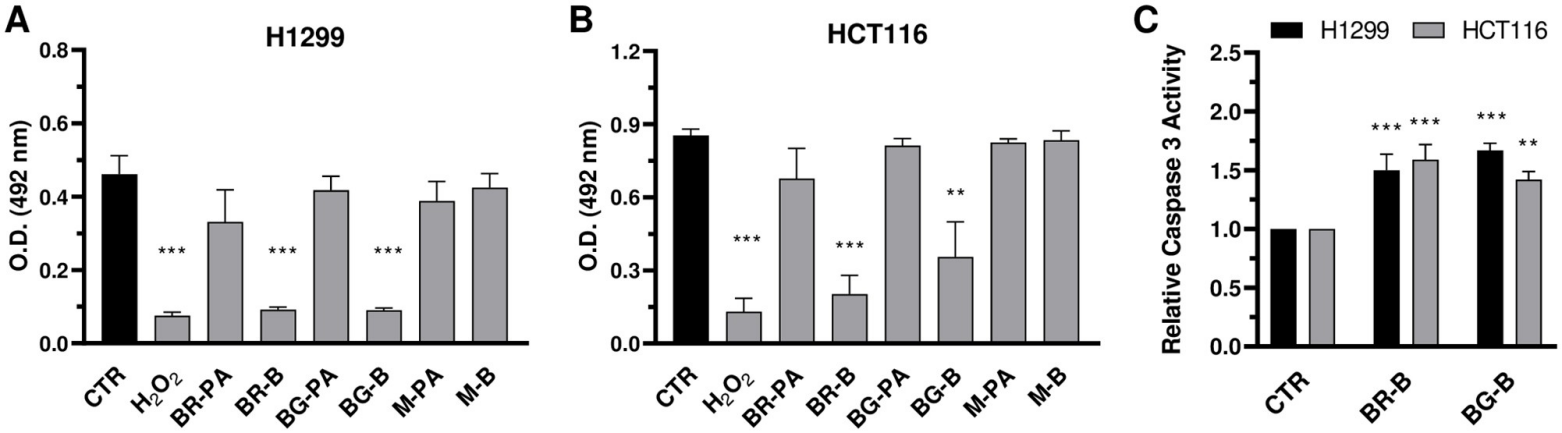
Characterization of cancer cell toxicity by different extracts from pomegranate immature and ripe fruits. Cytotoxicity after treatments with the indicated pomegranate fruit extracts (100 μg/mL for 24 h) was assessed by MTS in H1299 (*A*) and HCT116 (*B*) cells. O.D., optical density; H_2_O_2_, positive control (100 μM). (C) Caspase-3 activity induced by treatments with BR-B and BG-B (100 μg/ml for 24 h) was quantified with the Ac-DEVD-AMC substrate. Results are fold changes relative to the respective vehicle DMSO. *, p ≤ 0.05; **, p ≤ 0.01 and ***, p ≤ 0.001 vs respective vehicle DMSO. CTR, vehicle control (DMSO).

As a result, a significant inverse antiproliferative trend is present in peel extracts along the maturation process from BR to M (Fig 5A1), but not in mesocarp/aril extracts (Fig 5A2). These results reveal a previously unrecognized anticancer potential of immature baby red and baby green *P*. *granatum* fruits, and confer a yet unexploited value to these pre-harvest pomegranate byproducts from the thinning process. Compared with all other pomegranate extracts, BR-B induced the greatest, statistically significant anticancer effects in both tumor cell types, with the only exception of BG-B in H1299 cells (Table 2). BG-B, in turn, showed significantly reduced proliferative rates compared to all other extracts only in HCT116 (Table 2), suggesting possible cancer type specific susceptibility differences. To confirm occurrence of cancer cytotoxicity by pomegranate extracts, cell metabolic activity was assessed with MTS (Fig 6A and 6B). Treatments (100 μg/mL) with peel extracts from immature fruits, but not all other matrices, significantly suppressed cell metabolic viability compared to vehicle controls in both H1299 (Fig 6A) and HCT116 (Fig 6B) cells. Cytotoxic effects by BR-B and BG-B were of similar extent as those of cell damaging agent H_2_O_2_ (positive control; Fig 6A and 6B), and reflected induction of apoptosis through elevation of caspase-3 activity (Fig 6C). These observations shed light into the underlying molecular mechanism by which peel extracts from immature baby red and baby green *P*. *granatum* fruits might exert their anticancer effects in lung and colon adenocarcinomas, and are in agreement with those previously obtained with different ripe pomegranate matrices [11, 30]. Indeed, pomegranate tannin and punicalagin extracts induced apoptosis in HT29 [31] and Caco2 colon cancer cells through downregulation of anti-apoptotic bcl-XL and activation of caspase 3 [30]. Moreover, pomegranate leaf extracts and punicalagin exhibited selective cytotoxicity, cell cycle arrest and apoptosis in A549 [32] and H1299 lung cancer cells, in part, through activation of the mitochondrial intrinsic pathway [11]. Altogether, these findings indicate that peels from immature pomegranate fruits, especially ‘baby red’, are rich sources of natural metabolites with antitumorigenic activities. The exact chemistry of immature pomegranate constituents responsible for antitumor actions remains unresolved, warranting future in depth investigations on a wide range of possible causative metabolites [33–35]. However, considering the chemical composition of pomegranate extracts elucidated in this study, it is possible to speculate that granatins might substantially contribute to those anticancer effects. In fact, granatins were among the most abundant metabolites in peels, and showed an inverse accumulation gradient from BR-B to BG-B and M-B that mirrows the antiproliferative trend by pomegranate peel extracts in malignant cells (Fig 5A1). Moreover granatin B, the most abundant granatin present in immature peel extracts (Fig 3), has been recently demonstrated to exert apoptotic effects in glioblastoma cells [36], and could play similar roles in lung and colon adenocarcinoma cells (Fig 6C). Therefore, granatin B represents a promising candidate for future translational investigations aimed at developing novel prevention strategies against lung and colon cancers in the general population.

## Conclusions

This study defined the compositional landscapes of immature pomegranate fruits, cumbersome side products generated from pomegranate thinning procedures. These waste matrices (particularly peels) were much richer sources of polyphenols (especially gallotannins and ellagitannins) than ripe pomegranate fruits. Importantly, immature fruit extracts exhibited significant inhibition of proliferative kinetics in both human lung and colon cancer cells, wherein elicited specific induction of cytotoxicity and cell death mechanisms. Immature pomegranate fruit peels showed the highest antitumorigenic activity, possibly reflecting elevated gallotannin granatins content, a promising groundwork for future translational investigations. Therefore, pre-harvest *P*. *granatum* byproducts from the thinning procedure have great potential as nutraceutical/food additives and might harbor unexpected commercial opportunities for virtuous circular economy.

## Supporting information

S1 Fig Chemical structures of the pomegranate metabolites cited in this study. Please see Results & discussion for further details.
(DOCX)

S2 Fig Opposite accumulation trends observed for punicalagins (a and b) and gallotannins during fruit maturation (from BR to M). BR-PA, extract from ‘baby red’ immature pomegranate mesocarp and arils; BR-B, extract from ‘baby red’ immature pomegranate peels; BG-PA, extract from ‘baby green’ immature pomegranate mesocarp and arils; BG-B, extract from ‘baby green’ immature pomegranate peels; M-PA, extract from ripe pomegranate mesocarp and arils; M-B, extract from ripe pomegranate peels. See S1 Table for individual quantitative data.
(DOCX)

S3 Fig Inhibition of cancer cell proliferation by pomegranate immature and ripe fruits extracts. *Top panel*, representative microscopy images of human cancer H1299 and HCT116 cells treated for 48 h with the indicated pomegranate extracts (100 μg/mL). Images (10X magnification) were acquired with a light microscope (Evos XL, Thermo Fisher Scientific). CTR, vehicle control; scale bars, 400 μm. *Bottom panel*, bar graph of antiproliferative effects (means +/- SEM of data expressed as % of respective vehicle controls) from experiments (done in quadruplicate and repeated 3 times) illustrated in *top panel*. *, p ≤ 0.05; **, p ≤ 0.01 and ***, p ≤ 0.001 *vs* respective vehicle controls.
(DOCX)

S1 Table Metabolite contents (mg/g of fresh material) in the pomegranate matrices investigated. (DOCX)
