# Supplementary material for: Secondary metabolic profiles and anticancer actions from fruit extracts of immature pomegranates
Source: PLoS One. 2021 Aug 10;16(8):e0255831. doi: 10.1371/journal.pone.0255831 (PMC8354431; doi:10.1371/journal.pone.0255831)
Supplement: S1 Fig — Please see Results & discussion for further details. (DOCX) [file pone.0255831.s001.docx]

**S1 Fig**. Chemical structures of the pomegranate metabolites cited in this study. Please see Results & Discussion for further details.
