# Supplementary material for: Secondary metabolic profiles and anticancer actions from fruit extracts of immature pomegranates
Source: PLoS One. 2021 Aug 10;16(8):e0255831. doi: 10.1371/journal.pone.0255831 (PMC8354431; doi:10.1371/journal.pone.0255831)
Supplement: S2 Fig — BR-PA, extract from ‘baby red’ immature pomegranate mesocarp and arils; BR-B, extract from ‘baby red’ immature pomegranate peels; BG-PA, extract from ‘baby green’ immature pomegranate mesocarp and arils; BG-B, extract from ‘baby green’ immature pomegranate peels; M-PA, extract from ripe pomegranate mesocarp and arils; M-B, extract from ripe pomegranate peels. See S1 Table for individual quantitative data. (DOCX) [file pone.0255831.s002.docx]

**S2 Fig**. The opposite accumulation trend observed for punicalagins (a+b) and gallotannins during fruit maturation (from BR to M). BR-PA: extract from mesocarp and arils of “baby red” immature pomegranate; BR-B: extract from “baby red” immature pomegranate peels; BG-PA: extract from mesocarp and arils of “baby green” immature pomegranate; BG-B: extract from “baby green” immature pomegranate peels; M-PA: extract from ripe pomegranate mesocarp and arils; M-B: extract from ripe pomegranate peels. See Table S1 for individual quantitative data and text for further details.
