# Supplementary material for: Secondary metabolic profiles and anticancer actions from fruit extracts of immature pomegranates
Source: PLoS One. 2021 Aug 10;16(8):e0255831. doi: 10.1371/journal.pone.0255831 (PMC8354431; doi:10.1371/journal.pone.0255831)

**S3 Fig**. Inhibition of cancer cell proliferation by pomegranate immature and ripe fruits extracts. *Top panel*, representative microscopy images of human cancer H1299 and HCT116 cells treated for 48 h with the indicated pomegranate extracts (100 μg/mL). Images (10X magnification) were acquired with a light microscope (Evos XL, Thermo Fisher Scientific); CTR, vehicle control; scale bars, 400 µm. *Bottom panel,* bar graph of antiproliferative effects (means +/- SEM of data expressed as % of respective vehicle controls) from experiments (done in quadruplicate and repeated 3 times) illustrated in *top panel*. *, p ≤ 0.05; **, p ≤ 0.01 and ***, p ≤ 0.001 *vs* respective vehicle controls.


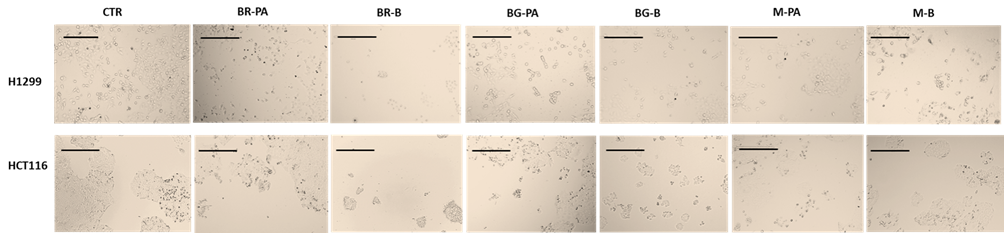


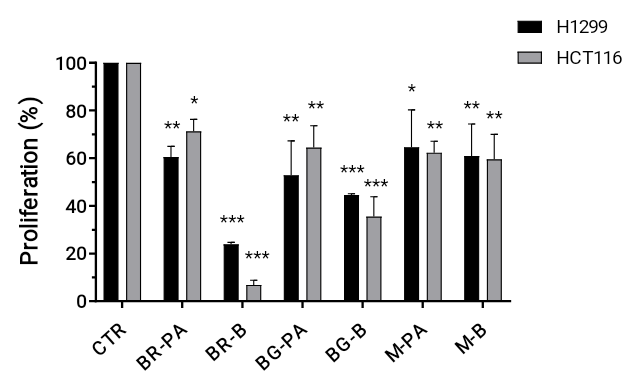

Supplement: S3 Fig — Top panel, representative microscopy images of human cancer H1299 and HCT116 cells treated for 48 h with the indicated pomegranate extracts (100 μg/mL). Images (10X magnification) were acquired with a light microscope (Evos XL, Thermo Fisher Scientific). CTR, vehicle control; scale bars, 400 μm. Bottom panel, bar graph of antiproliferative effects (means +/- SEM of data expressed as % of respective vehicle controls) from experiments (done in quadruplicate and repeated 3 times) illustrated in top panel. *, p ≤ 0.05; **, p ≤ 0.01 and ***, p ≤ 0.001 vs respective vehicle controls. (DOCX) [file pone.0255831.s003.docx]
