# Supplementary material for: Secondary metabolic profiles and anticancer actions from fruit extracts of immature pomegranates
Source: PLoS One. 2021 Aug 10;16(8):e0255831. doi: 10.1371/journal.pone.0255831 (PMC8354431; doi:10.1371/journal.pone.0255831)
Supplement: S1 Table — (DOCX) [file pone.0255831.s004.docx]

**S1 Table**. Content of individual metabolites from the pomegranate matrices object of this study, reported in mg over gram of fresh vegetable material. BR = “baby red” immature fruits; BG = “baby green” immature fruits; M = ripe pomegranate fruits; PA = mesocarp and arils; B = peels. See Figure 2, Figure 3 and text for details.

| **metabolite content, mg/g fresh vegetable material^a^** | | | | | | | |
| --- | --- | --- | --- | --- | --- | --- | --- |
|  | | **matrix^b^** | | | | | |
| Peak # | **compound** | BR-PA | BG-PA | BR-B | BG-B | M-B | M-PA |
| 1 | lagerstannin C (galloyl-HHDP-glucose) | 0,057 | 0,037 | n.d. | n.d. | n.d. | 0,007 |
| 2 | HHDP hexoside | n.d. | 0,062 | 0,570 | 0,885 | 0,151 | 0,023 |
| 3 | galloyl hexoside | 0,065 | 0,049 | 0,252 | 0,595 | 0,301 | 0,039 |
| 4 | galloyl-HHDP hexoside | 0,118 | 0,130 | n.d. | 0,139 | 0,653 | 0,072 |
| 5 | gallic acid | 0,185 | 0,052 | 0,829 | 0,537 | 0,021 | 0,001 |
| 6 | punicalagin derivative | 0,010 | 0,015 | n.d. | n.d. | 0,159 | 0,006 |
| 7 | punicalin (gallagyl hexoside) | 0,038 | 0,068 | 1,016 | 0,999 | 0,398 | 0,034 |
| 8 | punicalin isomer | 0,040 | 0,073 | 1,298 | 1,090 | 0,621 | 0,043 |
| 9 | di(HHDP-galloylglucose) pentoside | 0,804 | n.d. | 3,303 | n.d. | n.d. | n.d. |
| 10 | punicalagin isomer | n.d. | 0,219 | 5,949 | 1,200 | 2,095 | 0,091 |
| 11 | pedunculagin isomer 1 | 1,296 | n.d. | 12,144 | 21,344 | n.d. | n.d. |
| 12 | galloyl-hexoside isomer | 0,323 | 0,104 | n.d. | n.d. | n.d. | n.d. |
| 13 | digalloyl hexoside | 0,091 | n.d. | n.d. | n.d. | n.d. | n.d. |
| 14 | punicalagin a | 4,187 | 6,563 | 42,104 | 77,957 | 16,167 | 1,740 |
| 15 | punicalagin isomer | n.d. | n.d. | 14,693 | n.d. | n.d. | n.d. |
| 16 | pedunculagin III | n.d. | 0,148 | 5,033 | 10,656 | 2,035 | 0,245 |
| 17 | pedunculagin isomer 2 | 12,360 | 4,974 | 17,166 | 13,035 | 0,419 | 0,073 |
| 18 | punicalagin b | 7,398 | 14,736 | 79,927 | 142,100 | 27,494 | 2,694 |
| 19 | punigluconin isomer | 3,578 | 2,720 | 17,913 | 11,428 | 2,947 | n.d. |
| 20 | ellagic acid deoxy-hexoside | n.d | n.d. | 0,017 | n.d. | n.d. | 0,001 |
| 21 | punigluconin | 1,454 | 0,327 | 3,564 | 2,360 | 0,727 | 0,039 |
| 22 | pedunculagin isomer 3 | 7,565 | 1,355 | 5,695 | 0,643 | n.d. | n.d. |
| 23 | pedunculagin II | 6,342 | 0,946 | 5,143 | 6,139 | 0,354 | 0,019 |
| 24 | pedunculagin isomer 4 | 7,290 | 1,736 | 10,058 | 9,950 | n.d. | 0,032 |
| 25 | pedunculagin isomer 5 | 1,534 | 0,211 | 2,852 | 4,387 | 0,179 | n.d. |
| 26 | ellagic acid hexoside | 0,304 | 2,171 | 1,916 | 4,525 | 3,316 | 0,340 |
| 27 | granatin B | 7,722 | 0,870 | 54,250 | 32,359 | 4,076 | 0,063 |
| 28 | granatin B isomer | n.d. | 0,218 | 5,510 | 4,219 | 0,771 | 0,036 |
| 29 | ellagic acid galloyl hexoside | n.d. | 0,668 | n.d. | n.d. | 0,112 | 0,018 |
| 30 | ellagic acid pentoside | n.d. | 0,013 | 0,123 | 0,313 | 1,221 | 0,052 |
| 31 | ellagic acid galloyl hexoside isomer | n.d. | 0,015 | n.d. | 0,316 | 1,476 | 0,062 |
| 32 | ellagic acid | 0,491 | 0,398 | 5,012 | 5,759 | 1,803 | 0,089 |
|  | ***total polyphenols*** | ***63,25*** | ***38,88*** | ***296,34*** | ***352,94*** | ***67,50*** | ***5,82*** |
|  | *punicalagin a+b* | **11,59** | **21,30** | **122,03** | **220,06** | **43,66** | **4,43** |
|  | *total gallotannins* | **43,06** | **12,85** | **84,52** | **82,10** | **7,79** | **0,55** |
|  | *total ellagic acid derivatives* | **0,80** | **3,27** | **7,07** | **10,91** | **7,93** | **0,56** |
|  | *granatins* | **7,72** | **1,09** | **59,76** | **36,58** | **4,85** | **0,10** |
|  | *total gallotannins (including granatins)* | **50,78** | **13,94** | **144,28** | **118,68** | **12,63** | **0,65** |
|  | *% punicalagin over total polyphenols* | **18,32** | **54,78** | **41,18** | **62,35** | **64,69** | **76,21** |
|  | *% gallotannins over total polyphenols* | **80,29** | **35,85** | **48,69** | **33,63** | **18,72** | **11,15** |

^a^ as mean of three replicates; ^b^ see material and methods
